# First contact physiotherapy: an evaluation of clinical effectiveness and costs

**DOI:** 10.3399/BJGP.2023.0560

**Published:** 2024-08-06

**Authors:** Nicola E Walsh, Serena Halls, Rachel Thomas, Alice Berry, Cathy Liddiard, Margaret E Cupples, Heather Gage, Daniel Jackson, Fiona Cramp, Hannah Stott, Paula Kersten, Justin Jagosh, Dave Foster, Peter Williams

**Affiliations:** Centre for Health and Clinical Research, University of the West of England, Bristol, UK.; Centre for Health and Clinical Research, University of the West of England, Bristol, UK.; Centre for Health and Clinical Research, University of the West of England, Bristol, UK.; Centre for Health and Clinical Research, University of the West of England, Bristol, UK.; Centre for Health and Clinical Research, University of the West of England, Bristol, UK.; Centre for Public Health, Queen’s University Belfast, Belfast, UK.; Surrey Health Economics Centre, University of Surrey, Guildford, UK.; Surrey Health Economics Centre, University of Surrey, Guildford, UK.; Centre for Health and Clinical Research, University of the West of England, Bristol, UK.; Centre for Health and Clinical Research, University of the West of England, Bristol, UK.; Health and Social Care, Canterbury Christ Church University, Canterbury, UK.; Director for the Centre for Advancement in Realist Evaluation and Synthesis, Canada.; Department of Mathematics, University of Surrey, Guildford, UK.; Department of Mathematics, University of Surrey, Guildford, UK.

**Keywords:** general practice, physiotherapy, delivery of health care, musculoskeletal diseases, opioids

## Abstract

**Background:**

First contact physiotherapy practitioners (FCPPs) are embedded within general practice, providing expert assessment, diagnosis, and management plans for patients with musculoskeletal disorders (MSKDs), without the prior need for GP consultation.

**Aim:**

To determine the clinical effectiveness and costs of FCPP models compared with GP-led models of care.

**Design and setting:**

Multiple site case-study design of general practices in the UK.

**Method:**

General practice sites were recruited representing the following three models: 1) GP-led care; 2) FCPPs who could not prescribe or inject (FCPPs-standard [St]); and 3) FCPPs who could prescribe and/or inject (FCPPs-additional qualifications [AQ]). Patient participants from each site completed outcome data at baseline, 3 months, and 6 months. The primary outcome was the SF-36 Physical Component Summary (PCS) score. Healthcare usage was collected for 6 months.

**Results:**

In total, 426 adults were recruited from 46 practices across the UK. Non-inferiority analysis showed no significant difference in physical function (SF-36 PCS) across all three arms at 6 months (*P* = 0.667). At 3 months, a significant difference in numbers improving was seen between arms: 54.7% (*n* = 47) GP consultees, 72.4% (*n* = 71) FCPP-St, and 66.4% (*n* = 101) FCPP-AQ (*P* = 0.037). No safety issues were identified. Following initial consultation, a greater proportion of patients received medication (including opioids) in the GP-led arm (44.7%, *n* = 42), compared with FCPP-St (18.4%, *n* = 21) and FCPP-AQ (24.7%, *n* = 40) (*P*<0.001). NHS costs (initial consultation and over 6-month follow-up) were significantly higher in the GP-led model (median £105.5 per patient) versus FCPP-St (£41.0 per patient) and FCPP-AQ (£44.0 per patient) (*P*<0.001).

**Conclusion:**

FCPP-led models of care provide safe, clinically effective patient management, with cost-benefits and reduced opioid use in this cohort.

## Introduction

General practice is experiencing unprecedented demand for appointments at a time when the number of fully qualified GPs is falling, part-time working is increasing, and average patient caseload is rising.^1^ The Additional Roles Reimbursement Scheme was introduced in 2019 with the intention of growing the capacity of the primary care workforce.^2^ First contact physiotherapy practitioners (FCPPs) were one of five professional roles initially identified for expedited implementation,^2^ in recognition of the growing demands musculoskeletal disorders (MSKDs) place on general practice, which account for up to 30% of consultations.^3^ FCPPs have an extended appointment time (normally 20 minutes) to assess, diagnose, and determine the most appropriate interventions and manage onward referral for patients without the prior need for GP consultation.^4^ Some FCPPs also have the capability to provide injection therapy, and following legislation change in 2013, licensed physiotherapists can independently prescribe, including, since 2015, some controlled drugs.^5^ By 2024, all adults in England consulting with a suspected MSKD should be offered a consultation with a FCPP within their local practice.^6^

Since its inception, local service evaluations indicate that FCPPs reduce the need for GP consultation, referral to secondary care services, and prescribed medications, while improving patient and staff satisfaction.^7^ The only large-scale evaluation of FCPP was conducted as part of an NHS England national pilot of the initiative and reported against pre-determined criteria including the following: re-consultation rates with the GP; improvements in patient symptoms at 3 months; provision of self-management and/or exercise advice for the condition; and impact on ability to work.^8^ Pre-determined criteria were largely successfully met, apart from limited information on presenteeism and the ability to work. While this evaluation provided important data on the potential of FCPP, there was no insight regarding longer-term clinical outcomes, use of healthcare resources, or differences in outcomes compared with traditional GP-led models of care.

The current study aimed to determine the impact of FCPP on clinical outcomes and healthcare resource use for 6 months post-consultation compared with GP-led models of care.

**Table:** How this fits in

Introducing first contact physiotherapy practitioners (FCPPs) into general practice provides access to expert skills in musculoskeletal disorders (MSKDs) and helps manage patient demand for appointments; MSKD consultations account for up to one-third of GP workload. This study found that FCPPs provide a safe, clinically effective, and cost-beneficial alternative to GP-led consultations. FCPPs also positively impact on medication use (including opioids) and patients improve quicker than those who have not initially consulted with GPs. Embedding FCPP as a standard model in general practice will provide benefits for patients and savings for the healthcare system while reducing the number of patients consulting GPs with MSKDs.

## Method

### Setting and practice recruitment

General practices across the UK were invited to participate either via expressions of interest in response to a previous survey regarding FCPP provision,^9^ or through advertisement via Clinical Research Networks. The aim was to recruit across all four nations, from a range of urban and rural areas, and differing levels of deprivation; deprivation index was based on practice report and confirmed by nationally available data.^10^^–^13

### Description of services

General practice study sites were categorised into the following three study arms, according to their existing service provision:

1. no FCPP service: MSKD management with GP-led consultation (‘GP’);
2. standard FCPP with no additional competencies for prescribing and/or injecting (‘FCPP-St’); and
3. FCPP with additional qualifications to prescribe and/or inject (‘FCPP-AQ’).

### Participant recruitment

Patients who attended appointments for MSKDs in the study sites were given recruitment materials by the clinician or an allocated practice staff member. They were invited to contact the study team for further information, or to express their willingness to participate. Volunteers were screened for eligibility.

The inclusion criteria were as follows: 1) patients consulting with a suspected MSKD episode, defined as any acute or chronic disorder related to the spinal or peripheral musculoskeletal (MSK) system; 2) patients not consulted for the same problem in preceding 3 months; and 3) patients aged ≥18 years. The exclusion criteria were as follows: 1) receiving palliative care; and 2) non-English speaking and unwilling to provide informed consent and communicate through an interpreter.

Eligible participants provided written, informed consent. Recruitment started in December 2019, slowed in January 2020, owing to the emerging COVID-19 pandemic, and paused in March 2020. Recruitment re-started under COVID-19 restrictions in July 2020 and ended in April 2022. Final assessments were completed in October 2022.

### Data collection

Information on age, gender, reason for consultation, MSK risk (using STarT MSK), education, and employment were collected by telephone at baseline (post-consultation). Participants were also asked about their consultation experience and any safety concerns (to be reported elsewhere). There were no notable differences across groups.

Questionnaires regarding Patient Reported Outcome Measures (PROMs) were posted to participants following initial consultation (baseline) and at 3 months and 6 months post-consultation. The questionnaires were self-completed and returned by post. The primary outcome measure was the change from baseline to 6 months in the SF-36 Physical Component Summary (PCS) score.^14^ Secondary clinical outcomes were SF-36 Mental Component Summary score; Musculoskeletal Health Questionnaire (MSK-HQ, total and physical); perceived safety of health care, using the healthcare experience in general practice survey, short form (Patient Reported Experiences and Outcomes of Safety in Primary Care; PREOS-PC Q5), on a 10-point scale: completely unsafe (0) to completely safe (10); and Roland–Morris Disability Questionnaire (for patients with low back pain). EQ-5D-5L, a generic measure of health-related quality of life, was gathered for use in the economic evaluation.^15^

### Sample size

The total participants required per arm was 181 across 39 sites. This was based on a non-inferiority margin of 2 units in SF-36 PCS scale,^14^ a minimal clinically important difference of 4 points^16^ and standard deviation (SD) 6.5,^17^ a one-sided *P* = 0.05 non-inferior hypothesis test, with 80% power, a design effect of 1.09 for a cluster size of 14 and an intraclass correlation coefficient (ICC) of 0.0075,^18^ and 20% attrition. COVID-19 impacted recruitment, so figures were revisited. Actual attrition rates were used (5%) and number of sites were increased (*n* = 46), which required a total sample size of *n* = 462 (*n* = 154 per arm).

### Data analyses

The primary outcome was the change in SF-36 PCS score from baseline to 6 months compared between arms, using a one-way analysis of variance; in case of difference, a post-hoc unpaired *t*-test was performed. Further comparisons were undertaken in the context of stepwise linear regression modelling, incorporating demographic and clinical data, including baseline SF-36 PCS score. Outcomes from baseline to 3 months are also reported.

### Economic analysis

The base case economic analysis adopted an NHS and social care perspective. Information on service use related to the MSK condition was gathered retrospectively by telephone interview at 3 months and 6 months, using a tailored version of the Client Service Receipt Inventory (CSRI).^19^ This included: NHS and private healthcare services (primary, community, accident and emergency [A&E], outpatient referrals, and inpatient stays) and social care. Unit costs^20^^,^^21^ were applied to service use and summed (months 1–6) at the participant level, including the cost of the index consultation (see Supplementary Information S1). Group costs were inspected and compared. Owing to the skewed nature of the total costs data, stepwise logistic regression was used to model the presence or absence of additional costs over and above the cost of the initial presentation, with service model as a dummy variable and baseline demographic and clinical factors as covariates. A societal perspective was included through consideration of self-reported days off work and inability to perform usual activities, and the private perspective through out-of-pocket expenditures.

Analyses were carried out using IBM SPSS Statistics (version 27). Database access can be requested via: http://researchdata.uwe.ac.uk/703.

## Results

A total of 426 participants were recruited from 46 general practices across the UK, with a range of deprivation indices and rural or urban locations. Of the 426 participants, there were 110 (25.8%) from GP-led care, 124 (29.1%) from FCPP-St, and 192 (45.1%) from FCPP-AQ. A total of 46 GP practices were involved: 13 GP-led care practices (with 1, 2, 2, 5, 6, 6, 7, 10, 11, 14, 14, 15, and 17 participants), 15 FCPP-St practices (with 1, 3, 3, 3, 4, 4, 5, 7, 7, 9, 9, 14, 15, 17, and 23 participants), and 18 FCPP-AQ practices (with 1, 1, 4, 4, 6, 8, 8, 9, 11, 12, 14, 15, 15, 16, 16, 16, 17, and 19 participants). The study completion rates in each arm for PROMs and CSRIs, along with attrition patterns, can be seen in Supplementary Table S1.

Mean age was 63 years (SD 13.2); 34.1% (*n* = 145) were male and 97.8% (*n* = 408) reported White ethnicity. There were no statistically significant differences in individual baseline demographics between arms. There was some discrepancy in practice-level deprivation across arms, with a higher representation of low deprived practices in the FCPP-St arm (Table 1). Data were returned at all three time points by 377 (88.5%) participants, including 320 (75.1%) who provided completed PROM and CSRI data. Details of attrition from the study are given in Supplementary Table S1.

**Table 1.** Baseline demographics: summary statistics with comparison of the three service models

	**Total ( *N* = 426 participants)**			**GP (*n*=110 participants)**			**FCPP-St (*n* = 124 participants)**			**FCPP-AQ (*n* = 192 participants)**			**Comparison test**

**Demographic feature**	***N***	**Mean min–max**	**Standard deviation**	***N***	**Mean min–max**	**Standard deviation**	***N***	**Mean min–max**	**Standard deviation**	***N***	**Mean min–max**	**Standard deviation**	**ANOVA**
**Age, years**		63.0			63.2			63.1			62.8		
	425^a^	21.1–94.1	13.2	109^a^	21.5–89.9	13.3	124	21.1–83.6	12.8	192	13.4–94.1	13.4	*P* = 0.962

	***N***	***n***	**%**	***N***	***n***	**%**	***N***	***n***	**%**	***N***	***n***	**%**	χ**^2^**

**Sex, male**	425	145	34.1	110	37	33.6	123	41	33.3	192	67	34.9	*P* = 0.953

**Ethnic group, White**	417	408	97.8	107	106	99.1	122	116	95.1	188	186	98.9	n/a^b^

**Education**	410			108			119			183			Kruskal–Wallis
Primary or secondary		101	24.6		26	24.1		29	24.4		46	25.1	
Further education		179	43.7		51	47.2		57	47.9		71	38.8	
Associate degree		12	2.9		4	3.7		4	3.4		4	2.2	
Bachelor’s degree		70	17.1		18	16.7		16	13.4		36	19.7	
Master’s degree		24	5.9		6	5.6		6	5.0		12	6.6	
Professional degree		20	4.9		3	2.8		5	4.2		12	6.6	*P* = 0.512
Doctorate		4	1.0		0	0.0		2	1.7		2	1.1	

**Employment status**	418			108			121			189			χ^2^
Employed full-time		109	26.1		26	24.1		31	25.6		52	27.5	
Employed part-time		68	16.3		19	17.6		24	19.8		25	13.2	
Voluntary worker or unemployed and seeking work or homemaker or carer		40	9.6		9	8.3		13	10.7		18	9.5	
Retired		201	48.1		54	50.0		53	43.8		94	49.7	*P* = 0.749

**Site Deprivation Index^c^**	***N* = 46**	***n***	**%**	***N* = 13**	***n***	**%**	***N* = 15**	***n***	**%**	***N* = 18**	***n***	**%**	**Kruskal–Wallis**

High		13	28.3		3	23.1		4	26.7		6	33.3	*P* = 0.500
Medium		16	34.8		6	46.2		3	20.0		7	38.9	
Low		17	37.0		4	30.8		8	53.3		5	27.8	

*ANOVA = analysis of variance. FCPP-AQ = first contact physiotherapy practitioners-advanced qualifications. FCPP-St = first contact physiotherapy practitioners-standard. n/a = not available.*

a *One participant did not provide their age.*

b *An expected cell count of <5 for three out of six cells, caused by scarcity of ethnic groups other than White; only nine among 426 participants (2.1%) precluded a valid comparison test.*

c *Based on highest, middle, and lowest thirds.*

Clinical data revealed no statistically significant differences between arms at baseline, except for the EQ-5D-5L (visual analogue scale [VAS]; better state of health reported in FCPP-St model) and for MSK-HQ total (a more desirable MSK status was indicated in FCPP-St model). Participants reported a range of peripheral and spinal diagnoses (up to two pain sites); given the previously reported high incidence of low back pain in primary care,^18^ a 24.9% (*n* = 106/426) prevalence was noted (Table 2).

**Table 2.** Baseline clinical summary for each of the three service models

**Clinical feature**	**Total (*N* = 426 participants)**				**GP (*n* = 110 participants)**				**FCPP-St ( *n* = 124 participants)**				**FCPP-AQ ( *n* = 192 participants)**				**Comparison test**
	***N***	***n***	**%**		***N***	***n***	**%**		***N***	***n***	**%**		***N***	***n***	**%**		χ**^2^**
**MSKD area included back**	426	106	24.9		110	20	18.2		124	33	26.6		192	53	27.6		*P* = 0.165
	***n***	**Mean**	**Standard deviation**	**Min–max**	***n***	**Mean**	**Standard deviation**	**Min–max**	***n***	**Mean**	**Standard deviation**	**Min–max**	***n***	**Mean**	**Standard deviation**	**Min–max**	**ANOVA**
**SF-36 PCS**	403	35.6	10.5	10.2–62.3	103	35.3	9.3	15.7–55.7	118	36.8	10.2	12.6 –57.0	182	35.0	11.3	10.2–62.3	*P* = 0.338
**SF-36 MCS**	403	49.1	10.9	13.7–69.4	103	47.0	12.4	13.7–64.7	118	50.5	10.1	21.0–69.3	182	49.4	10.4	20.4–69.4	*P* = 0.051
**EQ-5D-5L score (England)^a^**	423	0.709	0.230	−0.281–1.000	109	0.683	0.262	−0.281–1.00	123	0.749	0.183	0.21–1.00	191	0.698	0.235	−0.241–1.00	*P* = 0.062
**EQ-5D-5L VAS**	422	68.8	19.3	0–100	109	66.7	20.0	15–95	122	72.6	17.3	10–100	191	67.6	19.9	0–100	*P* = 0.036^b^
**MSK-HQ total**	414	33.8	10.4	5–54	106	32.1	10.2	8–53	123	35.5	9.2	9–54	185	33.5	11.1	5–54	*P* = 0.044^b^
**MSK-HQ Physical activity**	421	2.7	2.4	0–7	109	2.39	2.31	0–7	123	3.01	2.46	0–7	189	2.71	2.42	0–7	*P* = 0.145
**Roland–Morris^c^**	98	9.4	6.1	0–24	18	11.2	5.8	1–20	32	8.2	5.9	1–21	48	9.5	6.4	0–24	*P* = 0.253
**STarT MSK pain intensity (0 to 10 [worst])**	401	6.3	2.3	0–10	105	6.4	2.3	1–10	117	6.1	2.2	0–10	179	6.4	2.3	0–10	*P* = 0.441

*ANOVA = analysis of variance. FCPP-AQ = first contact physiotherapy practitioners-advanced qualifications. FCPP-St = first contact physiotherapy practitioners-standard. MCS = Mental Component Summary. MSK = musculoskeletal. MSKD = musculoskeletal disorder. PCS = Physical Component Summary. VAS = visual analogue scale.*

a *Devlin* et al*.22*

b *Difference between groups of statistical significance but not of clinical significance; identifying hierarchy FCPP-St > GP, FCPP-AQ.*

c *Only reported in relation to participants with a diagnosis involving back pain.*

### Outcomes analysis

The primary outcome variable was the change in SF-36 PCS score from baseline to 6 months; in an unadjusted analysis, no statistically significant difference was found between arms (Table 3). This was confirmed under linear regression, with a final model (*R*^2^ = 0.138, *n* = 332) predicting change = 15.074–0.333x (SF-36 PCS score at baseline) + 2.377 (if university educated) + 2.402 (if in full-time employment). Service model along with age at baseline, gender (male: yes/no), ethnic origin (White: yes/no), whether MSKD area at baseline included back (yes/no), whether MSKD area at baseline included knee or leg or hip or foot or ankle (yes/ no), and whether the presented MSK condition had affected employment or ability to perform usual activities (yes/ no) were not significant (see Supplementary Table S2).

**Table 3.** Primary and secondary outcome changes from baseline to 3 months and from baseline to 6 months (positive changes indicate improvement)

**Primary outcome**	**Time point**	**Total (*N*= 426 participants)**					**GP (*n*= 110 participants)**					**FCPP-St (*n*= 124 participants)**					**FCPP-AQ ( *n*= 192 participants)**					**Change: Comparison test**	**Improved: Comparison test**

	**Months**	***N***	**Improved *n*(%)**	**Change mean**	**Change SD**	**Change range**	***N***	**Improved *n*(%)**	**Change mean**	**Change SD**	**Change range**	***N***	**Improved *n*(%)**	**Change mean**	**Change SD**	**Change range**	***N***	**Improved *n*(%)**	**Change mean**	**Change SD**	**Change range**	**ANOVA**	χ**^2^**
**SF-36 PCS**	3	336	219 (65.2)	2.72	8.42	−32.27 to 36.28	86	47 (54.7)	1.87	8.18	−17.82 to 27.73	98	71 (72.4)	3.69	8.05	−15.19 to 23.70	152	101 (66.4)	2.58	8.78	−32.27 to 36.28	*P*= 0.332	*P*= 0.037^a^
	6	348	234 (67.2)	4.15	9.78	−38.86 to 35.54	89	57 (64.0)	4.12	9.70	−28.67 to 29.06	107	75 (70.1)	4.18	8.98	−20.90 to 27.00	152	102 (67.1)	4.15	10.42	−38.36 to 35.54	*P*= 0.999	*P*= 0.667

**Secondary outcome**																							

**SF-36 MCS**	3	336	160 (47.6)	−0.14	8.25	−24.14 to 27.52	86	46 (53.5)	0.68	8.50	−23.07 to 26.30	98	43 (43.9)	−0.23	8.07	−24.14 to 27.52	152	71 (46.7)	−0.54	8.23	−23.84 to 17.52	*P*= 0.542	*P*= 0.409
	6	348	170 (48.9)	−0.43	8.78	−32.12 to 32.59	89	46 (51.7)	0.66	10.31	−32.12 to 32.59	107	50 (46.7)	−1.05	7.93	−24.15 to 16.66	152	74 (48.7)	−0.64	8.37	−28.94 to 23.54	*P*= 0.370	*P*= 0.786

**EQ-5D-5L score (England)^b^**	3	362	185 (51.1)	0.0347	0.1662	−0.656 to 0.897	96	44 (45.8)	0.0370	0.1712	−0.400 to 0.897	102	56 (54.9)	0.0350	0.1549	−0.350 to 0.519	164	85 (51.8)	0.0331	0.1710	−0.656 to 0.732	*P*= 0.984	*P*= 0.429
	6	376	229 (60.9)	0.0483	0.1639	−0.525 to 0.897	95	56 (58.9)	0.0480	0.1793	−0.508 to 0.897	113	70 (61.9)	0.0370	0.1463	−0.525 to 0.519	168	103 (61.3)	0.0561	0.1665	−0.398 to 0.790	*P*= 0.630	*P*= 0.898

**EQ-5D-5L VAS**	3	361	170 (47.1)	0.96	14.01	−55 to 70	96	42 (43.8)	0.58	16.75	−55 to 70	99	48 (48.5)	1.49	11.99	−35 to 45	166	80 (48.2)	0.85	13.46	−50 to 40	*P*= 0.895	*P*= 0.745
	6	371	169 (45.6)	0.50	16.94	−67 to 76	94	40 (42.6)	0.82	19.19	−55 to 76	111	46 (41.4)	−1.05	15.70	−67 to 55	166	83 (50.0)	1.36	16.42	−65 to 55	*P*= 0.501	*P*= 0.298

**PREOS-PC Q5 (0 to 10 [best])**	3	337	87 (25.8)	−0.09	1.74	−9 to 6	90	21 (23.3)	−0.06	1.59	−6 to 4	91	24 (26.4)	−0.26	2.17	−9 to 6	156	42 (26.9)	−0.01	1.52	−7 to 5	*P*= 0.535	*P*= 0.817
	6	348	84 (24.1)	−0.22	1.89	−8 to 5	90	22 (24.4)	−0.14	1.52	−5 to 4	101	32 (31.7)	−0.18	2.22	−8 to 5	157	30 (19.1)	−0.29	1.97	−7 to 5	*P*= 0.825	*P*= 0.070

**MSK-HQ Total**	3	356	232 (65.2)	3.29	8.05	−25 to 32	93	58 (62.4)	2.66	7.89	−24 to 32	102	67 (65.7)	3.61	7.98	−14 to 30	161	107 (66.5)	3.47	8.22	−25 to 26	*P*= 0.667	*P*= 0.798
	6	367	256 (69.8)	4.78	8.67	−23 to 34	92	68 (73.9)	5.22	8.29	−23 to 34	113	74 (65.5)	4.78	8.86	−18 to 32	162	114 (70.4)	4.52	8.80	−21 to 26	*P*= 0.830	*P*= 0.415

**MSK-HQ Physical**	`	362	118 (32.6)	0.03	2.13	−7 to 7	96	25 (26.0)	−0.10	2.11	−7 to 7	102	34 (33.3)	−0.07	2.05	−7 to 5	164	59 (36.0)	0.17	2.20	−7 to 7	*P*= 0.520	*P*= 0.252
	6	371	125 (33.7)	0.13	2.19	−7 to 7	94	21 (22.3)	−0.09	1.99	−5 to 7	112	46 (41.1)	0.29	2.03	−5 to 7	165	58 (35.2)	0.15	2.40	−7 to 7	*P*= 0.462	*P*= 0.016^a^

**Roland-Morris^c^**	3	72	38 (52.8)	−1.36	3.42	−10 to 6	11	5 (45.5)	−1.09	3.18	−7 to 3	23	11 (47.8)	−1.17	3.96	−10 to 6	38	22 (57.9)	−1.55	3.20	−7 to 4	*P*= 0.882	*P*= 0.650
	6	73	44 (60.3)	−1.95	3.72	−10 to 8	13	10 (76.9)	−2.62	2.72	−9 to 1	25	12 (48.0)	−1.20	4.31	−10 to 8	35	22 (62.9)	−2.23	3.59	−10 to 4	*P*= 0.449	*P*= 0.204

*ANOVA = analysis of variance. FCPP-AQ = first contact physiotherapy practitioners-advanced qualifications. FCPP-St = first contact physiotherapy practitioners-standard. MCS = Mental Component Summary. MSK = musculoskeletal. PCS = Physical Component Summary. VAS = visual analogue scale.*

a *Identifying hierarchy (FCPP-St, FCPP-AQ) > GP.*

b *Devlin* et al.*22*

c *Only reported in relation to participants with a low back pain-related diagnosis.*

However, when each of these change outcomes was simplified from the change in continuous score to an improved or worsened/stayed the same scenario, a statistically significant difference between arms was seen in two instances. At 3 months, the FCPP-St and FCPP-AQ service models delivered a statistically significant greater improvement rate for the primary outcome variable SF-36 PCS score compared with the GP-led service model (*P* = 0.037). At 6 months, the FCPP-St and FCPP-AQ service models delivered a statistically significant greater improvement rate for the secondary outcome MSK-HQ physical compared with the GP-led service model (*P* = 0.016; Table 3). No other statistically significant differences in outcomes were found between arms. No safety issues were identified.

### Healthcare utilisation and costs

The initial consultation was assumed to be face-to-face with a GP, FCPP-St, or FCPP-AQ. CSRI data were available for 370/426 (86.9%) of participants at 3 months, 348 (81.7%) at 6 months (see Supplementary Table S1). Health service use after the initial consultation was low in all arms, most being within general practice; few participants reported hospital use. Key health service usage (GP and physiotherapist) and prescribing outcomes are shown in Table 4*.* In the 3 months following initial consultation, a greater proportion of patients received medication (including opioids) in the GP-led arm (44.7%; *n* = 42) compared with FCPP-St (18.4%; *n* = 21) and FCPP-AQ (24.7%; *n* = 40) (χ^2^ *P*<0.001)*.* A full breakdown of NHS service use, including medication prescribing, at 3 months and 6 months, is shown in Supplementary Tables S3 and S4. There was scattered use of the private sector while use of over-the-counter medications was commonplace (see Supplementary Tables S5 and S6).

**Table 4.** Key self-reported NHS service usages associated with the presenting musculoskeletal condition, not including initial presentation, at 3 months and 6 months

**NHS service**	**3 months: Total (*N* = 370)**			**6 months: Total ( *N* = 348)**			**3 months: GP (*n* = 94)**			**6 months: GP ( *n* = 90)**			**3 months: FCPP-St ( *n* = 114)**			**6 months: FCPP-St ( *n* = 107)**			**3 months: FCPP-AQ ( *n* = 162)**			**6 months: FCPP-AQ ( *n* = 151)**		

	**Users, *n***	**%**	**Contacts (average)**	**Users, *n***	**%**	**Contacts (average)**	**Users, *n***	**%**	**Contacts (average)**	**Users, *n***	**%**	**Contacts (average)**	**Users, *n***	**%**	**Contacts (average)**	**Users, *n***	**%**	**Contacts (average)**	**Users,*n***	**%**	**Contacts (average)**	**Users, *n***	**%**	**Contacts (average)**
**General practice**																								
GP	52	14.1	74 (0.20)	30	8.6	39 (0.11)	29	30.9	47 (0.50)	14	15.6	21 (0.23)	10	8.8	11 (0.10)	8	7.5	9 (0.08)	13	8.0	16 (0.10)	8	5.3	9 (0.06)
Physiotherapist	90	24.3	135 (0.36)	38	10.9	69 (0.20)	9	9.6	18 (0.19)	5	5.6	14 (0.16)	27	23.7	43 (0.38)	11	10.3	26 (0.24)	54	33.3	74 (0.46)	22	14.6	29 (0.19)

**Outpatient referrals^a^**																								
Physiotherapy	38	10.3	80 (0.22)	26	7.5	60 (0.17)	16	17.2	42 (0.45)	8	9.0	17 (0.19)	11	9.6	20 (0.18)	8	7.5	19 (0.18)	11	6.8	18 (0.11)	10	6.6	24 (0.16)

**Prescribed medications**	**Users, *n***	**%**		**Users, *n***	**%**		**Users, *n***	**%**		**Users, *n***	**%**		**Users, *n***	**%**		**Users, *n***	**%**		**Users, *n***	**%**		**Users, *n***	**%**	

Any	103	27.6		66	19.0		42	44.7^b^		27	30.0^c^		21	18.4		16	15.0		40	24.7		23	15.2	
Analgesics	15	4.1		13	3.7		7	7.4f		7	7.8		2	1.8		2	1.9		6	3.7		4	2.6	
NSAIDs	43	11.6		23	6.6		16	17.0		8	8.9		7	6.1		7	6.5		20	12.3		8	5.3	
Opioids	53	14.3		36	10.3		27	28.7^d^		15	16.7^e^		6	5.3		7	6.5		20	12.3		14	9.3	

*FCPP-AQ = first contact physiotherapy practitioners-advanced qualifications. FCPP-St = first contact physiotherapy practitioners-standard. NSAIDs = non-steroidal anti-inflammatory drugs.*

a *Outpatient Referrals data missing for one GP participant.*

b *GP statistically significant (*P*<0.001) higher prevalence when compared with FCPP-St, FCPP-AQ, or to FCPP-St and FCPP-AQ combined at same time point.*

c *GP statistically significant higher prevalence when compared with FCPP-St (*P *= 0.011), FCPP-AQ (*P *= 0.006), or to FCPP-St and FCPP-AQ combined (*P *= 0.002) at same time point.*

d *GP statistically significant higher prevalence when compared with FCPP-St (*P*<0.001), FCPP-AQ (*P *= 0.001), or to FCPP-St and FCPP-AQ combined (*P*<0.001) at same time point.*

e *GP statistically significant higher prevalence when compared with FCPP-St (*P *= 0.025) or to FCPP-St and FCPP-AQ combined (*P *= 0.022), but not to FCPP-AQ (*P *= 0.088) at same time point.*

Group mean total costs (health services, excluding medications) over 6-month follow-up for the three service models are shown in Table 5*.* Comparisons were performed both excluding and including inpatient (planned MSK surgery) events, and assuming the FCPP-St and FCPP-AQ were both working at salary level band 7; a sensitivity analysis was performed with the FCPP-AQ costed at the higher band 8a. In each comparison, there is a statistically significant difference in costs between the three models (*P*<0.001) with the GP model the more costly (median £105.5 per patient versus £41.0 for FCPP-St and £44.0 for FCPP-AQ in the band 7 calculation), and no statistically significant difference between the FCPP-St and FCPP-AQ. In the band 8a comparison, the FCPP-AQ was significantly more costly than the FCPP-St. Regarding days lost through inability to work or perform usual activities, the FCPP-St model showed greater reductions in days lost compared with GP-led care and FCPP-AQ, but there was no statistically significant difference between GP-led care and FCPP-AQ (Table 6). Only eight participants had absences covered by sick notes in the first 3 months and three during the second period (two of which were new).

**Table 5.** Total costs (£) summary statistics, months 0–6

**Cost (£)**	**Total ( *N* = 425 participants)**				**GP (*n* = 109 participants)**				**FCPP-St ( *n* = 124 participants)**				**FCPP-AQ ( *n* = 192 participants)**				**Comparison test**

	***n***	**Mean**	**Median**	**Min–max**	***n***	**Mean**	**Median**	**Min–max**	***n***	**Mean**	**Median**	**Min–max**	***n***	**Mean**	**Median**	**Min–max**	**Kruskal–Wallis**
**Total excluding inpatient (FCPP-AQ band 7)**	348	142.77	52.00	22–1964	90	235.56	105.50	39–1738	107	112.95	41.00	22–952	151	108.59	44.00	22–1964	*P*<0.001
**Total including inpatient (FCPP-AQ band 7)**	348	382.47	52.00	22–16 784	90	507.44	105.50	39–16 334	107	260.92	41.00	22–16 784	151	394.11	44.00	22–15 922	*P*<0.001

**Total excluding inpatient, assuming band 8a (not band 7) for FCPP-AQ**	348	144.97	52.00	22–1967	90	235.56	105.50	39–1738	107	112.95	41.00	22–952	151	113.66	50.00	25–1967	*P*<0.001
**Total including inpatient, assuming band 8a (not band 7) for FCPP-AQ**	348	384.66	52.00	22–16 784	90	507.44	105.50	39–16 334	107	260.92	41.00	22–16 784	151	399.16	50.00	25–15 925	*P*<0.001

*FCPP-AQ = first contact physiotherapy practitioners-advanced qualifications. FCPP-St = first contact physiotherapy practitioners-standard. Initial consultation costs were: GP £39; FCPP-St £22; FCPP-AQ band 7 £22; and FCPP-AQ band 8a £25. All unit costs are given in Supplementary Information S1. The costs of medications and private treatment were excluded, as were the costs of wellness and exercise classes, and additional expenses, such as home help, personal care, home adaptations, mobility equipment, and transport for treatment costs, as these were extremely rare and reporting was patchy, so considered potentially unreliable.*

**Table 6.** Changes in days lost (unable to work or perform usual activities), with comparisons of the three service models

**Employment or usual activities**	**Time point**	**Total ( *N* = 426 participants)**				**GP (*n* = 110 participants)**				**FCPP-St ( *n* = 124 participants)**				**FCPP-AQ ( *n* = 192 participants)**				**Comparison test**

	**Months**	***N***	***n***	**%**	**Mean, median (IQR)**	***N***	***n***	**%**	**Mean, median (IQR)**	***N***	***n***	**%**	**Mean, median (IQR)**	***N***	***n***	**%**	**Mean, median (IQR)**	**Kruskal–Wallis**
**Change in days lost compared with pre-baseline**	3	284			−5.0, 0 (−2–0)	74			5.7, 0 (0–0)	81			−16.5, 0 (−10.5–0)	129			−3.9, 0 (−2–0)	*P* = 0.019
More			37	13.0			14	18.9			5	6.2			18	14.0		
Same			173	60.9			46	62.2			51	63.0			76	58.9		*P* = 0.049
Fewer			74	26.1			14	18.9			25	30.9			35	27.1		

**Change in days lost compared with months 0–3**	6	264			−8.7, 0 (−7–0)	68			−3.9, 0 (−7–0)	77			−20.5, 0 (−75–0)	119			−3.8, 0 (−3–0)	*P* = 0.063
More			26	9.8			7	10.3			5	6.5			14	11.8		*P* = 0.200
Same			154	58.3			42	61.8			42	54.5			70	58.8		
Fewer			84	31.8			19	27.9			30	39.0			35	29.4		

*FCPP-AQ = first contact physiotherapy practitioners-advanced qualifications. FCPP-St = first contact physiotherapy practitioners-standard. IQR = interquartile range. A negative number indicates fewer days lost. Mann–Whitney* U*test, pairwise comparisons of changes: 3 months: GP versus FCPP-St,* P *= 0.005; GP versus FCPP-AQ,* P *= 0.127; and FCPP-St versus FCPP-AQ,* P *= 0.093. 6 months: GP versus FCPP-St,* P *= 0.055; GP versus FCPP-AQ,* P *= 0.978; and FCPP-St versus FCPP-AQ,* P *= 0.031.*

Backwards stepwise logistic regression to model the presence or absence of additional health service costs in months 0–6 over and above the initial presentation (excluding inpatient), with re-running of the final model to include additional participants for whom data were missing only for non-significant predictors, led to the model in Supplementary Table S2 (with Nagelkerke *R*^2^ = 0.072, *n* = 334). The model demonstrates a significantly (2.181 times) higher likelihood of incurring additional costs after the initial consultation with a GP-led service model compared with a FCPP-St or FCPP-AQ service model. Higher scores in baseline SF-36 PCS score are also significantly associated with a lower likelihood of incurring additional cost (adjusted odds ratio of 0.966 implies that a participant with a baseline SF-36 PCS score, which is 10 points higher than another participant, is 0.966^10^ = 0.708 times less likely to incur additional cost). No other predictors were statistically significant.

The analysis demonstrated that neither FCPP model was inferior in relation to clinical outcome at 6-month post-consultation compared with the GP-led model, but both were significantly less costly; *P*<0.001. There were no significant differences in quality-of-life changes (based on EQ-5D-5L) between the models at 3 months or 6 months, so given the cost differentials, no formal cost-effectiveness analysis was undertaken (Tables 3 and 5).

## Discussion

### Summary

Analysis demonstrated no statistically significant difference in clinical outcomes between different service models after 6 months. However, the GP-led model of care was approximately 2.5 times costlier than the FCPP-St and FCPP-AQ models. Furthermore, at 3 months, a greater proportion of patients who consulted with FCPPs had improved, compared with those who had consulted with GPs, and time off work or unable to perform usual activities was reduced in the FCPP-St consultees.

### Strengths and limitations

To the authors’ knowledge, this is the first study that has compared GP-and FCPP-led models of care for MSKDs and included data from all four UK nations. It provides a robust overview of the service innovation to support decision making, and a qualitative analysis, which was conducted concurrently, will allow further interpretation of findings.

Recruitment was severely hampered by the COVID-19 pandemic, yet this study still provides the most extensive dataset of FCPPs to date. There was uneven recruitment across study arms and sites because the drive for FCPP recruitment, resulting from the Additional Roles Reimbursement Scheme, made the identification of GP-led sites challenging; and recruitment within some individual sites was lower than anticipated. At site level, there was some variation in deprivation across arms: the FCPP-St consisted of relatively more practices with lower levels of deprivation compared with the other arms, which may explain the higher levels of quality of life (EQ-5D-5L [VAS] and MSK-HQ) reported at baseline within this arm. However, while these differences were of statistical significance, neither was of clinical significance, based on previously reported levels of minimum clinical important difference^23^^,^^24^ and, importantly, there was no difference in the primary outcome measure at baseline across arms. All sites that expressed an interest in participation were recruited, so this variation did not result from selective recruitment. Furthermore, at the level of individual participants, no significant differences were found between groups regarding levels of education or employment.

The sample was almost exclusively White and not representative of practice cohorts despite efforts for diverse recruitment at practice and patient level. Only 12/46 (26.1%) sites returned requested data regarding numbers invited to participate in the study, so how representative the study sample is of those eligible is unable to be reported. Much of the recruitment was undertaken under COVID-19 restrictions, which disproportionately impacted people of ethnic minority heritage, which may have influenced decision to participate, although in consultation with recruitment sites, it was identified that fewer people from ethnic minority communities consult FCPP staff. There was potential recruitment bias as not all eligible participants consented to join the study.

### Comparison with existing literature

To the authors’ knowledge, this is the first study to show a comparison between GP and FCPP clinical outcomes and resource use, confirming the proposed benefits of the new model of care. While at 6 months there were no differences in patient improvement across the models studied, at 3 months a significantly greater proportion of patients who consulted with FCPPs had improved compared with GP consultees, with positive impact on ability to work or perform usual activities in FCPP-St (*P* = 0.005). Previous work highlighted GP propensity for pharmacological management rather than guideline-based self-management and rehabilitation strategies, which may account for these differences;^25^^–^27 indeed, a greater proportion of patients under GP-led care were prescribed medication, including opioid derivatives. The authors are unable to identify any factors in the study design that would account for this finding and believe this is a result of clinical decision making. Other work has shown that FCPPs with a licence to prescribe are still reluctant to use this intervention, instead choosing to use their capability to deprescribe where possible and intervene with non-pharmacological measures.^28^

From an onward resource use perspective, data showed minimal reliance on other services within each model and therefore relatively low costs. For services that were used, there was a greater number of referrals onto outpatient physiotherapy by GPs, as would be expected; other work has suggested GP overuse of magnetic resonance imaging (MRI), but this was not found.^29^ These data were obtained through self-report so may have been subject to recall bias. It is noted, however, that other studies report the similarities in self-report versus medical record review, and in some cases note greater accuracy with patient recall.^30^

A previous evaluation in England reported that GP workload was positively impacted by FCPPs. It found most patients did not consult their GP with the same problem within 3 months of seeing the FCPP.^8^ This concurs with the present study’s findings that only 23/276 (8.3%) of patients consulted the GP for the same problem having seen the FCPP, whereas many more (30.9%) initial GP consultees re-consulted the GP for the same problem within the study period (Table 4).

A predominant aim of introducing FCPPs is to make better use of resources in general practice. The present study shows clear cost benefits to implementing FCPP models compared with GP-led care given the extent of MSKD consultations in primary care.^3^

### Implications for research and practice

This research supports continued implementation of FCPP in general practice as a safe, clinically effective, and cost-beneficial approach to managing people with MSKDs. Given FCPPs’ low reliance on prescription medications, it may also assist in reducing opioid prescriptions in primary care. Further research is required to understand why there appears to be disproportionate consultations from people of ethnic minority heritage to ensure appropriate access for all.

## Data

University of the West of England Database Repository Database access can be requested via: http://researchdata.uwe.ac.uk/703.
